# Emotional Burden and Perceived Social Support in Male Partners of Women with Cancer

**DOI:** 10.3390/ijerph17124188

**Published:** 2020-06-12

**Authors:** Marcin J. Jabłoński, Francisco García-Torres, Paulina Zielińska, Alicja Bułat, Piotr Brandys

**Affiliations:** 1 Institute of Psychology, Faculty of Philosophy, Jesuit University Ignatianum in Krakow, 31-501 Kraków, Poland; marcinjablonski@interia.pl; 2 Department of Psychology, University of Cordoba, 14071 Cordoba, Spain; 3 IMIBIC Health Research Institute, Reina Sofía University Hospital of Cordoba, 14004 Cordoba, Spain; 4 The Maria Skłodowska Curie Memorial Cancer Centre and Institute of Oncology (MCMCC) branch in Krakow, 31-115 Kraków, Poland; pzielinska29@gmail.com (P.Z.); alivia1909@gmail.com (A.B.); z5brandy@wp.pl (P.B.)

**Keywords:** male caregiver, emotional burden, perceived social support, cancer, patients’ distress anxiety, and depression

## Abstract

Background: The aim of this study was to describe the correlations between the psychosocial burden on male caregivers and their perception of social support, as well as distress, anxiety, and depression among their partners in the first six months after a cancer diagnosis. Methods: A cross-sectional, longitudinal and observational study was conducted on a group of 61 couples, with the use of Zarit Burden Interview (ZBI), Caregiver Burden Scale (CBS), Berlín Social Support Scales (BSSS), Hospital Anxiety and Depression Scale (HADS) and Distress Thermometer (DT). Statistical analysis was performed using Statistica v.13. Results: A strong positive correlation between the ZBI and CBS, as well as between support-seeking and the emotional involvement of male partners, was documented. The negative correlation between the lack of instrumental support and a much greater burden on caregivers, in emotional, social, and family life was documented. The level of distress, anxiety, and depression, as well as family problems reported by female patients, were positively correlated with the male caregiver′s burden. A demographic analysis showed significant relationships between the number of offspring and the negative health indicators of patients and their partners. Implications: The obtained results encourage deeper reflection on the need to improve the availability of instrumental support for male caregivers and support for families with an oncological ill parent in caring for minor children, and to maintain the social activity of the caregiver.

## 1. Introduction

A family caregiver is broadly defined as a relative or friend who provides unpaid assistance to a person with a chronic or disabling condition, during a significant amount of time, and frequently without previous training [1,2]. These new tasks may negatively influence different areas including conflict with personal and family relations, less physical and psychological health with a reduced quality of life, and the interference or change in work status as a consequence of the assumption of the caregiving role [3,4]. Family caregivers play an essential role in assisting cancer patients at home, but systematic assessments of their needs are rarely carried out [5]. Providing support as an informal caregiver may result in caregiver burden. The caregiver burden can be defined as the perception of the degree to which their physical health and psychological well-being, social life and financial status are affected by the patient’s illness [6]. Previous research with other chronic illness, such as Alzheimer′s disease, have shown that gender is a risk factor for perceived health in caregivers [7]. We know much about caregiving women compared with caregiving men [8], but there is low information however, about the reactions of male caregivers in terms of burden. While not conclusive, research increasingly suggests that women caregivers experience more burden than men [4], but in recent decades, the gender composition of family caregivers who provide unpaid informal care to persons with medical illness has changed noticeably: male caregivers were 25% of the caregivers surveyed in 1987 and were 40% in 2016 [9], so research on men in the context of their care tasks is gaining in importance. Male caregivers have been characterized as more instrumental, focused on specific tasks in contrast with female caregivers who also tend to the emotional work, maintaining identities and relationships [9]. Male caregivers, as a separate group with their own needs, have not received much attention in the cancer literature, and their concerns and challenges may differ from those of female caregivers [10,11]. A recent study has provided evidence that the husbands of patients with cancer reported strains concerning a social environment, and in sexual, vocational, domestic, and extended family relations, and that coping in this group of caregivers is different when compared with husbands of people without cancer [10]. Some studies find greater psychological distress levels among husbands than is found in their wives with cancer [11]. However, there is little research on the experience of male caregivers in the care of cancer partners in the context of their specific needs for social support, in the first moments of cancer diagnosis and little is known about how caregivers’ experiences change over time [10,12]. Therefore, this study attempts to obtain information about the burden (general, emotional and social) of male caregivers in the context of caring for patients with different kinds of cancer, shortly after the close relative cancer diagnosis (45–60 days after) of the disease, and to establish the relations between the burden of male caregivers and their perception of social support, and the degree of distress, anxiety and depression among their female partners within this period.

## 2. Materials and Methods

### 2.1. Participants

This was a cross-sectional, longitudinal, and observational study. Participants (couples) were recruited in the order of admission of women with cancer to the oncology unit in Krakow, Poland, from January to December 2018. The inclusion criteria were: (1) men aged 18–65, currently in intimate relationships with women (aged 18–65) who were diagnosed with cancer before 30–45 days, and who were qualified for further surgery, chemotherapy, or radiation therapy; (2) inclusion in the study takes place in the order of applications to the Oncology Center; (3) all participants should be mentally able to answer the questionnaire, as determined by the psychologist at the first meeting; (4) the caregiver and patient must live in the same household; (5) partners who do not work professionally as a guardian of a sick person; and (6) persons who have signed informed consent. At each stage of the project, each participant has the right to withdraw consent from continued participation in the project, for any reason. Information about the withdrawal will be recorded in the documentation of the study. A similar situation will occur in the case of withdrawal of consent (or death) of the person with cancer disease. 

### 2.2. Instruments

Previous research has not used consistent approaches to measure caregiver burden, and there are substantial variations in the types of survey questions about caregiving experiences [12]. That is why we decided to use the two most common scales for assessing caregiver burden: the Zarit Burden Interview (ZBI) is widely used to assess burden in cancer settings and has good psychometric properties [13,14]. It has 22 items, ranging from 1 (never) to 5 (nearly always), in which higher scores indicate a higher burden. Following the recommendations of the MAPI Research Trust, and following the proposals presented by Whitlatch et al. [15], we distinguished the following dimensions of caregiver burden: burden in the relationship, emotional burden, social and family life burden, financial burden, loss of control over one’s life, personal strain and role strain. The degree of burden can be estimated by the sum of the points obtained, as little or no burden (0–20 points, degree of burden = 1), mild to moderate burden (21–40 points, degree of burden = 2), moderate to severe burden (41–60 points, degree of burden = 3), or severe burden (61–88 points, degree of burden = 4).

The Caregiver Burden Scale (CBS) developed by Elmstahl et al. [16]. The CBS consists of 22 questions divided into five factors: general strain, disappointment, emotional involvement, environment, and isolation. Each question has four response alternatives: “not at all” (scored as 1), “seldom” (scored as 2), “sometimes” (scored as 3), and “often” (scored as 4). The mean of all the answers gives the total burden score in the range of 22–88 points. A higher score indicates a greater burden. The CBS has satisfactory validity and reliability with kappa values in the range of 0.89–10.0 [17,18], and has been used in psycho-oncology [19]. A Polish version of this scale is available [20,21].

Participants also completed the following scales. The Berlín Social Support Scales (BSSS) [22,23] is a tool is dedicated to measuring the cognitive and behavioral aspects of social support. The scales were developed for and validated with an adult population of cancer patients and their partners. It is a self-administered tool. Participants indicate their agreement with the statements and the answering format is the same for all subscales: patients rate their agreement with the statements on a four-point scale. Possible endorsements are strongly disagree (1 point), somewhat disagree (2 points), somewhat agree (3 points), and strongly agree (4 points). The BSSS describes the following dimensions of support for a person in need of support in 5 subscales: perceived social support, need for support, support seeking, received social support, and protective buffering. In our study, we focused on the first three (BSSS I–III) of these five dimensions. The validity of BSSS has been demonstrated in several studies [22]. The scale has been used in previous studies involving the Polish population [24,25,26,27]. The instrument the Distress Thermometer (DT [28,29] was developed to be applied in cancer settings, and it is an easy measure of distress, consisting of a line with a 0 (no distress) to 10 (extreme distress) scale. The patient marks their perceived levels of distress on the scale answering the question: “How distressed have you been during the past week on a scale 0–10? “. The scale also has a problem checklist in different domains (practical problems: range 0–5 points, family problems: 0–2 points, emotional problems: 0–6 points, spiritual/religious concerns: 0–1 point, and physical problems: 0–21 points). A score above 3 on the analog scale is considered as a measure of elevated distress [29]. Hospital Anxiety and Depression Scale (HADS) [30] is an instrument commonly used in oncology settings to assess anxiety and depression in two different scales (anxiety and depression) with seven items scoring from 0 to 3, and the maximum score is 21. A score above 8 is considered a good cut-off point for both anxiety and depression, and it is validated in the Polish population with good psychometric properties [31]. Finally, all the participants completed a questionnaire on clinical and sociodemographic data including age, body mass, marital status, number and age of children, living situation, length of the relationship, place of residence, amenities (elevator, pram ramps in the apartment, school, pharmacy, public transport near the place of residence, own car), level of education, employment status, chronic diseases, level of recreational physical activity and addictions.

### 2.3. Procedure

The participants were approached by psychologists during the patient’s clinic visit. Following informed consent and personal instruction, all the assessment questionnaires were completed either at the clinic or at home and returned by mail. The patients and caregivers were interviewed separately. The first measurement (T1) was done 45–60 days from the diagnosis. For the couple, this was based on demographic data, for the male caregivers, on ZBI, CBS and BSSS, and for female patients, on DT and HADS. The second measurement (T2) was carried out six months later (caregivers: ZBI, CBS, and BSSS, patients: DT and HADS). All subjects gave their informed consent for inclusion before they participated in the study. This study was conducted in accordance with the Declaration of Helsinki, and the protocol was approved by the Bioethics Committee of the Regional Medical Chamber in Kraków No. 151/KBL/OIL /2016. STROBE checklist was completed (Strengthening the Reporting of Observational Studies in Epidemiology (See Table S1.)).

### 2.4. Statistical Analysis

Statistical analysis was performed using Statistica v.13.1 (StatSoft Polska Sp. z o.o, Kraków, Poland). The relationships between the studied variables at T1 were checked using Spearman’s correlations. Under Stanisz’s recommendation [32], the following scale for assessing the correlation was adopted: *r_s_* in the range of 0.3–0.5—average, *r_s_* in the range of 0.5–0.7—high, *r_s_* in the range of 0.7–0.9—very high. When the *r_s_* value was less than 0.3, the correlations were regarded as very weak and not considered. To check for differences between the scales between the first (T1) and second tests (T2), the Wilcoxon pair order test was used. Statistical significance was assumed at the level *p* < 0.05.

## 3. Results

In this study, sixty-one pairs (122 participants) were qualified and tested at T1 using the instruments previously described. The second observation (T2) included 34 pairs. After T1, 20 pairs withdrew from the study without giving a reason, two patients died, one couple separated, and four relocated for further treatment. The age of the women with cancer ranged from 22 to 67 years (*M* = 43.6), 41 of them (680.3%) had a diagnosis of breast cancer, and the remaining 20 (32.7%) had cancer in different locations (head and neck: n = 6, digestive tract: n = 2, reproductive organs: n = 2, connective tissue and skin: n = 10). Surgery as the only method of treatment was used in 31 cases (50.8%), and 30 women (49.2%) underwent combined therapy (surgery with chemotherapy and/or radiotherapy). Thirty-four women (55.7%) declared recreational physical activity at least once a month, 13 (210.3%) once a week, and 3 (5%) daily. The other 11 women (18%) declared no such activity. Only four patients (6.5%) smoked cigarettes. Fifty-four women (88.5%) scored more than 80 points on the Barthel Index of Activities of Daily Living (BI-ADL), which means that they were independent in everyday life, and the remaining seven patients (11.5%) scored in the range of 60–79 points, which corresponds to the “minimally dependent” functional state. The sociodemographic data of male partners as caregivers are presented in Table 1.

The general results of the ZBI, CBS, and BSSS in the male partners of the women with cancer are presented in Table 2. The overall results of the burden were little or moderate, depending on the questionnaire and the different subscales. In terms of social support, the general scores were high, while the scores of distress using the analog scale of DT showed significant values of severe distress. Finally, the scores of anxiety reached clinically relevant values, but the scores of depression were low.

The strong positive correlation between the ZBI and CBS was documented (*r_s_*(61) = 0.57, *p* < 0.001). The relationship between the burden on male caregivers and the perceived social support is presented in Table 3.

Only one positive correlation was observed between seeking support and the emotional involvement of the male partners. There was a negative correlation between the lack of instrumental support and a much greater burden on caregivers in the emotional, social, and family life dimension of ZBI, and higher ratings in the disappointment and environment subscales, as well as in the total burden in CBS. When looking for a correlation between the burden on a partner and the emotional condition of treated women, anxiety and depressive symptoms and the distress level in cancer patients were taken into account. It was observed that the total level of distress, anxiety and depression symptoms, as well as family problems reported by the female patients, were positively correlated with the caregiver’s burden. The results are shown in Table 4. 

The present study showed the strongest correlation of the analogue DT scale with the number of emotional (*r_s_*(61) = 0.56, *p* < 0.001) and physical (*r_s_*(61) = 0.57, *p* < 0.001) problems, as well as the sum of all problems (*r_s_*(61) = 0.57, *p* < 0.001) listed in the descriptive part of the test. There were no significant correlations between the severity of distress, anxiety and depression in the female patients and the perceived social support in their partners. The analysis of the demographic variables showed significant relationships between the number of offspring and the negative health indicators of patients and their partners. The number of children positively correlated with the level of distress (*r_s_*(61) = 0.28, *p* = 0.02), anxiety (*r_s_*(61) = 0.26, *p* = 0.04) and depressive symptoms (*r_s_*(61) = 0.38, *p* = 0.002) in female cancer patients. The number of children up to 10 years of age positively correlated with the number of practical problems (*r_s_*(61) = 0.27, *p* = 0.03) and family problems indicated by women in the descriptive part of the DT (*r_s_*(61) = 0.39, *p* = 0.02), with a greater burden on male partners in social and family life in ZBI, and also with the dimension of isolation of male caregivers in CBS. In contrast, the number of teenagers (10–19 years) positively correlated with the level of disappointment (*r_s_*(61) = 0.32, *p* = 0.01) and the lower perception of instrumental support in CBS in men. Interestingly, the number of adult children in the family did not affect reducing the burden on fathers. 

In the longitudinal setting, six months after the first examination (T2), the results obtained from 34 pairs were analyzed. Significant differences in the Distress Analogue Scale were observed in treated women in the Wilcoxon pair order test. Interestingly, in parallel with lower distress (in T1: *M(SD)* = 60.06(2.7), median = 6.50 vs. T2 = *M(SD)* = 4.62(2.75), median = 50.00, *p* = 0.02), women reported more problems in the functioning of the family (average T1 = 0.15 vs. T2 = 0.56, *p* = 0.02). There were no significant differences in patients’ HADS scores. There were no significant changes in the burden and perceived social support among male partners. In the observation, Spearman’s rank correlation method showed a positive correlation between the number of children and persistent emotional problems in DT (*r*_s_(61) = 0.37, *p* = 0.02) and the severity of depression in HADSD (*r*_s_(61) = 0.44, *p* = 0.009) in female patients. On the other hand, in male partners, the number of children positively correlated with a greater burden in relations (*r*_s_(61) = 0.38; *p* = 0.02) and a greater personal burden (*r*_s_(61) = 0.37, *p* = 0.03).

## 4. Discussion

Most research on caregiver burden relates to the advanced or terminal period of cancer [33,34]. Little is known, however, about the burden on caregivers at the beginning of the disease, and as we know, this period is a serious source of emotional crisis in the patient′s family. Johansen et al. [35] carried out an interesting cross-sectional study in which they investigated 281 patient–caregiver couples in which one partner had different kinds of cancer (breast, prostate, head and neck cancers, as well as melanoma and lymphoma) at the beginning or in an early stage of the patients’ radiation treatment. Depression, fatigue, energy levels, and symptoms in patients were not significantly associated with caregiver burden [35]. In contrast to these findings, in our study, the total levels of distress, anxiety, and depression symptoms, as well as family problems reported by the female patients, were positively correlated with the male caregiver′s burden. From the consultative point of view, it is interesting that it is not the female partner′s cancer itself and its somatic consequences, but her mental condition that is an important source of burden on the male caregiver. This could result in additional recommendations for the implementation of active screening and the treatment of emotional problems in oncologically ill patients. Other authors reported varying amounts of role strain in the husbands of women with breast cancer, with the highest being in the social environment, followed by sexual, vocational, domestic, and extended family relations [36]. Grunfeld et al. [34] used the ZBI to study 89 caregivers of women with advanced breast cancer at the start of the palliative period. The mean score of the caregiver burden in this study was 180.3 and did not change significantly during the entire palliative period. This result, which corresponds to a mild–moderate degree of burden, is similar to our data. In a study by Garlo et al. [33], the overall level of burden showed only minimal change over the 12 months of observation, and the relationship between the time and burden did not remain significant in multivariable analysis. This is consistent with our study, which also did not show significant changes in the burden among male partners in the one-year follow-up. The caregivers of patients with acute lymphoblastic leukemia reported high caregiver burden, and the burden was higher among caregivers who reported lower perceived social support [37]. Moreover, the Garlo’s study, which used a short form of ZBI, revealed that the heavy burden on cancer patients’ caregivers was associated with caregiver reports about the need for more help in daily tasks, but not with objective measures of the patient′s need for assistance [33]. This may suggest a lack of appropriate perceived instrumental support in this group, and lead to the conclusion, drawn by the authors, that the burden can be a measure of the caregiver′s ability to adapt to new tasks in a caring role. Data from other studies confirm that the lack of receipt of formal services by caregivers was associated with their greater burden [38]. This may be because caregivers most frequently identify support with technical daily tasks, as an area in which more support would be needed [39,40]. Other research results show that male caregivers, in particular, have been characterized as more instrumental, focused on specific tasks, in contrast with female caregivers [41]. This is also in line with our observation about the importance of instrumental support to lessen the male caregiver burden in many dimensions. In Garlo’s study, nearly all the caregivers who reported a need for more emotional support reported a high burden, compared to caregivers who did not need more support [33]. Our observations are similar. They show, in more detail, that there is a positive correlation between seeking support and the emotional involvement of male partners, and a negative correlation between the lack of instrumental support of a caregiver and his much greater emotional, social, and family burden. In Garlo’s analysis, there was no statistically significant correlation between the presence of depression in patients and the burden on caregivers. In contrast to these data, we obtained statistically significant correlations between these variables. This is consistent with the data of other authors, e.g., Lee et al. [42], who observed that the caregiver burden was higher among those whose recipients had greater symptom distress.

In a sociodemographic analysis of similar patient–caregiver dyads, other authors [11] found that, among spouses, those with more education tended to be more burdened, but our observations do not confirm these findings. The authors did not find earlier studies on caregiver burden in cancer patients that took account of the number of children in the family as a sociodemographic factor that may modify the burden level of male caregivers. The analysis of demographic variables showed significant relationships between the number of offspring and the negative health indicators of both patients and their partners, especially with the level of distress and depressive symptoms in female cancer patients. The number of children up to 10 years of age positively correlated with the number of practical problems and family problems indicated by women, and with a greater burden on male partners in social and family life and with their feeling of isolation. This condition can lead to a specific “vicious wheel of burden”, because, as described by other authors, the social isolation of caregivers is in itself a greater burden [5,43]. In contrast, the number of teenagers (10–19 years) positively correlated with the disappointment level and a lower perceived instrumental support in male caregivers. However, potential reasons for this correlation remain difficult to explain. The assumption that the stress of caregiving may provoke some kind of psychological regression, and redirect the male caregiver’s perception towards the perspective typical for teenagers they look after, seems far too notional. Finally, this research has some limitations that are necessary to mention. The study was cross-sectional and was carried out in a relatively small group of male partners of women with different kinds of cancer, and the data were collected at a single oncology center at the beginning of long-term treatment, and the number of pairs that did not last until the second observation was high. Thus, the associations found cannot serve as general conclusions. We also did not assess the role of religion and spirituality, which could bring additional valuable information and serve to discuss the topic more holistically. Due to this, the results of this study should be considered only as preliminary. Furthermore, more comprehensive and randomized research in a larger group could be necessary, taking into account these limitations.

### Clinical Implications

The presented results fit into the context of organizational changes in the Polish support system for family caregivers, focusing mainly on the caregivers of the disabled and the elderly. The proposed activities mainly include regular visits by a nurse specializing in in-home care [44,45]. We are deeply convinced that assessing the burden and needs of male caregivers from the perspective of perceived social support would help determine the appropriate support services, ensuring high-quality care, achieving satisfaction, and reducing the burden on caregivers. This can be the basis for the development of support standards for the caregiver, and for the education of medical staff in terms of a holistic view of the patient–caregiver pair in the context of optimizing therapeutic and educational activities. Practical information on the key importance of instrumental support and means of providing real help for children in families with cancer are concrete guidelines for the better organization of the social support system.

## 5. Conclusions

Our observations highlight the need to take a holistic approach and to recognize and address additional psycho-social concerns in families with cancer. In particular, these results should encourage specialists to deepen their reflection on the need to improve the availability of the extended instrumental support for male caregivers, and not just to mobilize their initial resources to support a partner with cancer. The practical support related to care for children in families with cancer, including helping caregivers maintain adequate social activity, seems to be an important task for a comprehensive healthcare system oriented towards the patient–partner dyad.

## Figures and Tables

**Table 1:** Sociodemographic data of the male partners of the women with cancer.

Description	
Sex: male (*N* = 61) Age Range: 24–67 (*M* = 45.8, *SD* = 9.7) Information about the Offspring	
Number of children: 0–5 (*M* = 1.7) Value (%)	
Childless	10 (16.4)
1 child	12 (19.7)
2 children	28 (45.9)
3 children	8 (13.1)
4 children	1 (1.6)
5 children	2 (30.3)
Young children (<10 years old)	25 (40.9)
Adult offspring (11–19 years)	23 (37.79)
Children in adulthood (>19 years old)	25 (40.9)
Place of residence	
Village	20 (32.8)
A city of up to 100,000	11 (18)
An average city of 100,000–300,000	4 (6.6)
Big city > 300,000	26 (42.6)
Duration of the relationship	
<5 years	9 (14.7)
6–10 years	8 (13.1)
11–15 years	12 (19.7)
16–20 years	8 (13.1)
21–30 years	17 (27.9)
31–40 years	7 (11.5)
Education	
Basic education	1 (1.6)
Vocational	13 (210.3)
Secondary	25 (41)
Higher	22 (36.1)
Subjective health assessment	
Average	7 (11.5)
Good	39 (63.9)
Very good	15 (24.6)
Chronic diseases	
Yes	15 (24.6)
No	46 (75.4)
Recreational physical activity	
None	12 (19.7)
Once a month	30 (49.2)
Once a week	14 (22.9)
Daily	5 (8.2)
Addictions	
Cigarettes	13 (21.4)
Alcohol	1 (1.6)
No	47 (77)
Own car	
Yes	56 (91.8)
No	5 (8.2)

**Table 2:** Results of the ZBI, CBS, and the BSSS in the male partners of the women with cancer.

Burden Dimensions	*M*	*SD*	Median	Minimum	Maximum
Zarit Burden Interview Results (ZBI)					
The burden in the relationship	5.41	2.53	50.00	0.00	10.00
Emotional burden	4.77	2.98	40.00	0.00	150.00
Social and family life burden	1.82	1.77	20.00	0.00	70.00
Financial burden	10.08	1.27	10.00	0.00	40.00
Loss of control over one′s life	40.34	2.41	40.00	0.00	110.00
Personal strain	9.28	4.77	90.00	10.00	230.00
Role strain	3.18	2.74	30.00	0.00	90.00
Total ZBI	170.38	80.37	170.00	30.00	440.00
Degree of burden	10.30	0.49	10.00	10.00	30.00
Caregivers Burden Scale results (CBS)					
General strain	15.57	4.84	150.00	80.00	280.00
Isolation	5.25	1.98	50.00	30.00	10.00
Disappointment	9.70	2.98	90.00	50.00	170.00
Emotional involvement	4.70	1.49	50.00	30.00	90.00
Environment	4.97	1.83	50.00	30.00	110.00
Total CBS	40.20	10.15	410.00	220.00	650.00
Berlin Social Support Scales results (BSSS)					
BSSS I - perceived emotional support	130.39	2.29	140.00	80.00	160.00
BSSS I - perceived instrumental support	140.08	2.18	150.00	90.00	160.00
BSSS I - perceived general support	27.48	4.10	280.00	170.00	320.00
BSSS II - need for support	11.15	2.43	110.00	40.00	160.00
BSSS III - support seeking	12.80	30.35	130.00	30.00	190.00
DT analogue scale	60.03	2.40	60.00	0.00	10.00
DT practical problems	0.67	10.04	0.00	0.00	40.00
DT family problems	0.21	0.45	0.00	0.00	20.00
DT emotional problems	2.77	1.49	30.00	0.00	60.00
DT spiritual/religious	0.03	0.18	0.00	0.00	10.00
DT physical problems	5.21	3.53	50.00	0.00	140.00
HADS-A	80.05	4.72	80.00	10.00	180.00
HADS-D	50.00	3.97	40.00	0.00	160.00

**Table 3:** Correlations of the caregiver burden (ZBI and CBS) and the perceived social support (BSSS) in the group of male partners of the women with cancer.

Burden Domains		Perceived Social Support/BSSS	*r_s_*	*p*
ZBI	Emotional burden	Instrumental support	−0.39	0.002
		Emotional support	−0.30	0.019
		General support	−0.36	0.004
	Social and family life burden	Instrumental support	−0.30	0.020
		General support	−0.31	0.014
	Personal strain	Need for support	−0.30	0.020
	Role strain	General support	−0.32	0.010
CBS	Disappointment	Emotional support	−0.40	0.001
		Instrumental support	−0.33	0.010
		General support	−0.40	0.001
	Emotional involvement	Support seeking	0.30	0.019
	Environment	Emotional support	−0.36	0.004
		Instrumental support	−0.36	0.004
		General support	−0.39	0.002
	CBS total	Emotional support	−0.35	0.006
		Instrumental support	−0.30	0.017
		General support	−0.37	0.003

ZBI—Zarit Burden Interview, CBS—Caregivers Burden Scale, BSSS—Berlin Social Support Scales.

**Table 4:** Analysis of the correlation between the burden of the male caregivers, anxiety, and depression, and stress in a woman with cancer.

Male Caregiver Burden		DT and HADS Scores in a Woman with Cancer	*r_s_*	*p*
ZBI	Degree of burden	Family problems listed in DT	0.36	0.004
	Social and family life burden	Family problems listed in DT	0.32	0.012
	Loss of control over one’s life	DT analogue scale	0.39	0.002
		Distress general *	0.31	0.015
		HADS-A	0.30	0.017
	Role strain	HADS-D	0.32	0.013
CBS	General strain	DT analogue scale	0.33	0.010
		Distress general	0.32	0.013
		HADS-A	0.33	0.009
	Disappointment	DT analogue scale	0.31	0.016
	Emotional involvement	Emotional problems listed in DT	0.33	0.010
	Environment	Family problems	0.37	0.003
	Total burden	DT analogue scale	0.36	0.004
		Distress general	0.32	0.012
		HADS-A	0.31	0.016
		HADS-D	0.30	0.019

ZBI—Zarit Burden Interview, CBS—Caregivers Burden Scale, DT–Distress Thermometer, HADS–Hospital Anxiety (A) and Depression (D) Scale, * Distress general = sum of all the identified problems listed on the Distress Thermometer.

## Data Availability

The original research documentation is stored in the archives of the Maria Skłodowska-Curie Institute of Oncology in Kraków. The data that support the findings of this study are available from the corresponding author upon reasonable request.

## Supplementary Materials

The following are available online at https://www.mdpi.com/1660-4601/17/12/4188/s1, Table S1: STROBE Statement—checklist of items that should be included in reports of observational studies.
